# Rapid *in vivo* testing of drug response in multiple myeloma made possible by xenograft to turkey embryos

**DOI:** 10.1038/bjc.2011.445

**Published:** 2011-11-01

**Authors:** Y Farnoushi, M Cipok, S Kay, H Jan, A Ohana, E Naparstek, R S Goldstein, V R Deutsch

**Affiliations:** 1 Department of Hematology, Tel Aviv Sourasky Medical Center, Sackler Faculty of Medicine, Tel Aviv University, 6 Weizman Street, Tel Aviv 62439, Israel; 2 Mina and Everard Goodman Faculty of Life Sciences, Bar Ilan University, Ramat Gan, Israel

**Keywords:** multiple myeloma, turkey embryo xenograft, drug response

## Abstract

**Background:**

The best current xenograft model of multiple myeloma (MM) in immune-deficient non-obese diabetic/severe-combined immunodeficient mice is costly, animal maintenance is complex and several weeks are required to establish engraftment and study drug efficacy. More practical *in vivo* models may reduce time and drug development cost. We recently described a rapid low-cost xenograft model of human blood malignancies in pre-immune turkey. Here, we report application of this system for studying MM growth and the preclinical assessment of anticancer therapies.

**Methods:**

Cell lines and MM patient cells were injected intravenously into embryonic veins on embryonic day 11 (E11). Engraftment of human cells in haematopoietic organs was detected by quantitative real-time polymerase chain reaction, immunohistochemistry, flow cytometry and circulating free light chain.

**Results:**

Engraftment was detected after 1 week in all embryos injected with cell lines and in 50% of those injected with patient cells. Injection of bortezomib or lenalinomide 48 h after cell injection at therapeutic levels that were not toxic to the bone marrow dramatically reduced MM engraftment.

**Conclusion:**

The turkey embryo provides a practical, xenograft system to study MM and demonstrates the utility of this model for rapid and affordable testing therapeutics *in vivo*. With further development, this model may enable rapid, inexpensive personalised drug screening.

Multiple myeloma (MM) is a disease characterised by proliferation and accumulation of malignant plasma cells in the bone marrow (BM), bone lesions and immunodeficiency, with immunoglobulin light chains usually detected in the blood. Although new treatment options are available, MM remains an incurable malignancy with a grim prognosis. Preclinical evaluation of activities of new drugs and their combinations *in vitro* and *in vivo* is critical for drug development. Practical *in-vivo* models for studying human MM may enable a better understanding of the biology of the disease and lead to better optimisation of therapeutic strategies. The best current xenotransplantation *in-vivo* model for studying human blood malignancies and therapy is the immune-deficient mice, including the severe-combined immunodeficient (SCID) mice (Yaccoby *et al*, 1998; Nefedova *et al*, 2008; Chauhan *et al*, 2010; Li *et al*, 2011); the non-obese diabetic/SCID (NOD/SCID) mice (Dazzi *et al*, 2000; Lock *et al*, 2002) or the even more permissive *β*-microglobulin-null (B2m−/−) NOD/SCID mice (Peled *et al*, 2002; Feuring-Buske *et al*, 2003; Pearce *et al*, 2006). Although the murine model can recapitulate the phenotype of human disease, immune-deficient mice are very costly, require special maintenance and the engraftment of human leukaemias and myeloma in mice requires from 6 weeks to over 2 months (Lock *et al*, 2002; Li *et al*, 2011), unless the cells are grafted directly to the BM using a technically demanding procedure with lower rates of survival (Yaccoby *et al*, 1998; Tassone *et al*, 2005).

Xenotransplantation of human cells and tissues to chicken embryos (Boulland *et al*, 2010) and particularly to the chorioallantoic membrane (CAM) is a well-established system for evaluating angiogenesis in solid tumour growth (Ribatti *et al*, 2006; Rebbaa *et al*, 2009). Solid tumours that are grafted to the CAM are known to reproduce many characteristics of tumours *in vivo*, including tumour mass formation, angiogenesis and metastasis (Koop *et al*, 1995; Kobayashi *et al*, 1998). In contrast to hundreds of studies in chicken embryos using solid tumours, engraftment of human blood malignancies on the CAM or on the haematopoietic organs has not been studied in depth. We have demonstrated consistent engraftment of the human leukaemia line K562 in the haematopoietic organs of chicken embryos 1–2 weeks after intravascular or intra-amnion injection (Taizi *et al*, 2006). However, the turkey embryo proved to be a more robust *in-vivo* system for the assessment of human leukaemia growth and response to doxorubicin (Grinberg *et al*, 2009).

In this study, we extended the development of this new model to studying MM, by transplanting human cell lines and patient samples in naturally immune-deficient turkey embryos and testing the effect of several new therapies. Similar to the model in immune-deficient NOD-SCID mice (Yaccoby *et al*, 1998; Tassone *et al*, 2005), we were able to demonstrate engraftment and response to therapy. However, unlike the NOD/SCID model, the turkey embryo model is inexpensive, easy to manipulate and requires no maintenance. Engraftment is rapidly detected 1 week after injection by flow cytometry (FC) and quantitative real-time polymerase chain reaction (Q-PCR) of BM cells and circulating free light chain (FLC) in the blood. We applied this model to test several drugs used as first-line therapy in MM, thalidomide, bortezomib (Velcade) and lenalinomide (Revlimid). Thalidomide has been widely used as front-line and relapse therapy to treat MM (Palumbo, 2010; Ghosh *et al*, 2011; Kumar *et al*, 2011). Although the exact mechanism of action in MM is not known, thalidomide induces oxidative stress, and it has both antiangiogenic and immunomodulatory activities (Melchert and List, 2007). Bortezomib is a proteasome inhibitor targeting the 26S proteasome, a component of the ubiquitin–proteasome complex (Zhang *et al*, 2009). Bortezomib directly inhibits proliferation and induces apoptosis of MM cells, as well as cells resistant to conventional therapies (Nefedova *et al*, 2008). Revlimid is a less toxic thalidomide derivative that has potent antitumour, antiangiogenic, immunomodulatory and proapoptotic activity in MM (Richardson *et al*, 2010). Although thalidomide proved to be too toxic for use in the developing embryos, a single dose of therapeutic levels of bortezomib and Revlimid dramatically inhibited MM cell engraftment in turkey embryos. We believe that these results support the use of this simple and inexpensive model system to provide a new platform for studying human MM *in vivo*, which can be easily employed for testing the efficacy of new anti-myeloma agents.

## Materials and Methods

### Cells and cultures

Human samples were obtained from patients with MM and control samples were obtained from patients undergoing orthopaedic surgery, following the signing of informed consent by all participants. The use of human material in this study was approved by the Tel-Aviv Sourasky Medical Center Ethics Committee according to the regulations of the Helsinki accords, and the DNA studies were approved by the Israel Ministry of Health. Peripheral blood and BM mononuclear cells were separated by Ficoll-density gradient centrifugation. Human cell lines CAG and ARH-77 were kindly donated by Dr B Katz of the Tel Aviv. ARH-77 were established from an Epstein–Barr virus (EBV)-positive plasma cell leukaemia patient, and CAG cells were derived from human MM cells and are EBV-negative (Borset *et al*, 2000). Cells were maintained in RPMI medium 1640 supplemented with 10% foetal bovine serum and 100 U ml^−1^ penicillin, and 100 mg ml^−1^ streptomycin (Biological Industries, Bet Haemek, Israel) and passaged twice weekly. Patient samples were taken from newly diagnosed untreated (*n*=4) and progressive disease or relapsed patients (*n*=10). Patient samples were taken from newly diagnosed untreated (*n*=4) and progressive disease or relapsed patients (*n*=10).

### Introduction of human myeloma cells into turkey embryos

Fertilised turkey (*Meleagris gallopavo*) eggs were obtained from a local supplier (Yeffe Hod, Kvuzat Yavneh, Israel). According to Israeli law, similar to the office of laboratory animal welfare of the US public health service, avian embryos are not considered live vertebrate animals until hatching. The NIH Office of Laboratory Animal Welfare has provided written guidance in this area (http://www.grants.nih.gov/grants/olaw/references/ilar91.htm and NIH Publication No.: 06-4515). Our study did not include turkey embryos incubated for more than 24 days. In turkeys, hatching occurs at 26–28 days of embryonic development; therefore, approval by an ethics committee was not necessary. Avian embryos greater than two-thirds of the way to hatching (in turkeys that is day 16) can experience pain and embryos beyond E15 (<2/3) were euthanised by decapitation as suggested in guidelines of San Francisco State University and Case Western University (http://research.sfsu.edu/protocol/policy_library/avian_embryo.html, http://casemed.case.edu/ora/iacuc/forms/Policy%20for%20Use%20of%20Avian%20Embryos.pdf). The eggs were incubated in a humidified egg incubator (GQF Manufacturing Company, Savannah, GA, USA) at 37.5°C, with turning. A small aperture was cut in the eggshell for injecting embryos, which was subsequently sealed with gas-permeable tape. Cells suspended in 100 *μ*l phosphate-buffered solution (PBS) were injected intravenously into the CAM vessels of E11 embryos, or at indicated developmental stages, as described previously (Taizi *et al*, 2006; Grinberg *et al*, 2009).

### Histology

Tissues from the injected turkey embryos were fixed in 4% paraformaldehyde or Bouin's solution, embedded in paraffin, sectioned and stained with hematoxylin and eosin (H&E) to observe tissue morphology. Photomicrographs were taken on fluorescence-equipped Olympus BX60 upright and SX10 stereomicroscopes using Scion monochrome and colour digital cameras (http://www.scion.com) and ImageJ (http://rsb.info.nih.gov/ij/). No digital modifications were made to the photos, with the exception of merging monochrome fluorescent images into colour images, and brightness/contrast improvement and sharpening were performed with the Paint Shop Pro (V.7.04) software (www.jasc.com) or a Microsoft picture editor.

### Detection of engrafted human cells by FC

Turkey embryos were killed on E18, or as indicated, and single-cell suspensions were prepared from the liver, spleen and BM (pooled from both femurs). The cells were analysed following lysis of enucleated red blood cells (RBCs) with FACS lysis buffer (BD Bioscience, San Diego, CA, USA). Bone marrow cell suspensions from embryos injected with ARH-77 or CAG cells were incubated with anti-human CD138-FITC (IQ products, Groningen, The Netherlands), which detects MM cells and anti-human CD19-APC (BD Biosciences), a B lymphocyte-lineage differentiation antigen. When patient samples were injected, BM cell suspensions from the grafted embryos were stained with anti-CD138-FITC, anti-PE-CD38 (BD), anti-CD45-perCP (BD) and anti-56APC (BD). Multiple myeloma cells from all patient samples injected were previously shown to be CD138 and CD38 positive and negative for CD45 and CD56. All incubations were for 30 min at 4°C. At least 10 000 events per sample were acquired with a BD FACS Calibur (BD Biosciences). Data analysis were performed using Cell Quest and Cell Quest Pro software (BD Biosciences). Matched isotype controls for all antibodies were used to detect background fluorescence (BD Biosciences). All human antibodies were pre-tested for crossreactivity with avian cells using BM from non-injected embryos.

### Molecular detection of xenografted human cells by Q-PCR

To detect human cells, the BM, liver, spleen and brain were harvested and DNA was extracted using phenol. DNA samples were incubated in Light Cycler DNA master SYBR green I (Roche Diagnostics GmbH, Mannheim, Germany), primers as described (10 *μ*M sense and 10 *μ*M antisense), MgCl_2_ and nuclease-free water in a total volume of 10 *μ*l according to the manufacturer's instructions. To estimate the amount of human cells that had engrafted in the various embryonic organs, Q-PCR was performed by developing a molecular assay to quantify the relative contributions of human and mouse in mixed DNA samples. DNA from CAG or ARH-77 cells was used for measuring human alpha satellite sequences. The avian *GAPDH* gene from turkey BM DNA of embryos was used for quantifying turkey cells. The sequences used to detect human alpha satellite and chicken *GAPDH* are listed in Table 1 (purchased from Danyel Biotech Ltd, Rehovot, Israel). The RT–PCR conditions used were 40 cycles consisting of 95°C for 10 min, 95°C for 20 s, 60°C for 20 s, 72°C for 30 s, 72°C for 5 s, 95°C for 5 s, 70°C for 15 s and 40°C for 30 s. Standard curves were generated using a series of 10-fold dilutions of chicken or human DNA as described previously (Taizi *et al*, 2006). The human primers did not generate amplification products using DNA from non-injected turkey embryo BM. Using 1.2 pg of DNA per CAG or ARH-77 cell and 2.6 pg of DNA per normal turkey cell, we calculated the ratio of human/chicken cells. The domestic turkey has recently been reported to have 80 chromosomes and the DNA content we obtained is comparable with that reported (Dalloul *et al*, 2011).

### Serum FLC

Free light chain levels in embryos injected with human myeloma cells were determined by automated assays for free *κ* and *λ* on a IMMAGE 800 nephelometer (Beckman Coulter Inc., San Francisco, CA, USA) using commercial reagents (The Binding Site, Birmingham, UK). This assay is routinely performed in our clinical laboratory for patient testing of human *κ* and *λ* light chains in the serum. The concentration of soluble FLC *κ* or *λ* in serum samples is determined by a turbidimetric assay. A series of calibrators of known antigen (FLC *κ* or *λ*) concentration are assayed to produce a calibration curve of measured light scatter *vs* FLC *κ* or *λ* concentration (http://www.bindingsite.co.uk) (Supplementary Figure 1S). The assay involves an addition of the test sample to solution containing the appropriate antibody in reaction cuvette. As the antigen–antibody reaction proceeds, the light from a beam passing through the cuvette is increasingly scattered by insoluble immune complexes that are formed. Light scatter is monitored by measuring the decrease in intensity of the incident beam of light. The antibody in the reaction is present in excess so that the amount of immune complex formed is proportional to the FLC *κ* or *λ* concentration. Samples of human controls (The Binding Site) were within the expected control range for FLC *κ* (13.28–19.92 mg l^−1^) and for FLC *λ* (21.28–31.92 mg l^−1^). Assays were performed at a 1 : 5 serum dilution with a detection range 3–90 mg l^−1^ according to the manufacturer's instructions. The FLC level in uninjected embryonic turkey serum samples (*n*=27) served as negative controls, with levels consistently below the detection range <3 mg l^−1^.

### Cell viability

The toxicity of drugs on the developing BM cells was determined by FC using 7-amino actinomycin D (7AAD) (Sigma-Aldrich, St Louis, MO, USA) to stain non-viable cells.

### Treatment of embryos with anti-myeloma drugs

Stock solutions of bortezomib (Velcade) (Millennium Pharmaceuticals Inc., Cambridge, MA, USA), thalidomide and lenalinomide (Revlimid) (Celgene Corp., Summit, NJ, USA) were dissolved in 70% ethanol/30% PBS and diluted in PBS for injection. All drugs were injected in a final volume of 100 *μ*l. Preliminary experiments were performed to determine non-lethal doses of bortezomib, thalidomide or Revlimid for the developing turkey embryos. Thalidomide was extremely toxic even at very low doses, consistent with its well-known teratogenic effects, and was therefore not used in this study. All of the embryos injected intravenously on E13 with 0.2 *μ*g per embryo of bortezomib or Revlimid survived for 7 days. In addition, no BM toxicity was observed using FC of cells stained with 7AAD at this dose. To test the effects of these drugs on engraftment *in vivo*, a single dose of 0.2 *μ*g per embryo of bortezomib or lenalinomide was injected intravenously on E13, 2 days after injection of the human cell lines into CAM veins of E11 embryos. After 6 days, the embryos were killed and the haematopoietic tissues were collected for DNA and FC analyses.

### Statistical analysis

The statistical significance of differences between test and control groups was determined using Student's *t*-test or analysis of variance.

## Results

### Engraftment of human MM into turkey embryos

**Figure 1:**
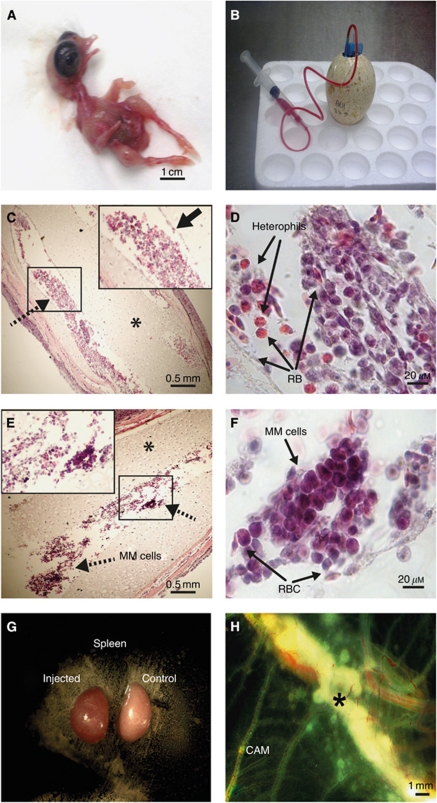
Engraftment of human myeloma cells in embryonic day 18 (E18) embryonic turkey bone marrow. (**A**) Turkey embryo on E18 with blood vessels large enough to enable drawing blood (**B**). (**C**–**F**) Paraffin sections of E18 femur bones stained with haematoxylin and eosin (H&E). (**C**) Haematopoiesis has clearly been established, with robust haematopoiesis seen in the developing bone marrow niches (**C** and **D)** ( × 4 original magnification indicated by dotted arrow in **C**). Stromal cells line the borders of the haematopoietic sinuses (solid black arrow in the inset in **C**). **(D**) A highly proliferative area within the indicated box at × 40 original magnification with nucleated RBC, immune heterophils and other haematopoietic cells, as well as defined borders of the haematopoietic sinus and prominent cartilage areas (indicated by an asterisk). (**E**) A typical femur from an E18 turkey embryo injected on E11 with ARH-77 multiple myeloma cells at × 4 original magnification. Bone marrow haematopoiesis is irregular and meager with large areas of discontinuous or missing stromal borders of the sinus (inset in **E**), with clusters of myeloma cells that can be noted (indicated by arrows) having typical eccentric nuclei and myeloma cell morphology. **(F**) Multiple myeloma growth seen at a higher magnification ( × 40 original magnification) with chondrogenesis and bone structure appearing normal. Photographs of the spleens of injected embryos were not enlarged on E18; however, they were consistently more red in colour (**G**). This is seen in the left spleen from an injected embryo compared with a normal embryonic spleen. Growths that were noted on the chorioallantoic membrane 1 week after injection contained both turkey and CAG cells, which continued to grow in size (**H**).

Multiple myeloma cell lines CAG or ARH-77 (5 × 10^6^) cells were injected into CAM vessels on day E11 and engraftment was detected 1 week later (E18) as described previously (Grinberg *et al*, 2009). A typical E18 embryo is shown in Figure 1A. Blood was collected (1.5–2 ml) from these embryos with a 23 G ‘butterfly’ needle (Figure 1B), and paraffin sections of the femur bone from non-injected (Figure 1C and D) and injected (Figure 1E and F) embryos were stained with H&E. The developing BM with prolific haematopoiesis could easily be observed in the control embryos. There was robust haematopoietic proliferation at E18 (Figure 1C), and the borders of the haematopoietic sinuses were clearly delineated (inset in Figure 1C), with early and mature nucleated RBC, prominent heterophils and other haematopoietic and mesenchymal cells (Figure 1D). Cartilage areas and chondrocytes, which are spatio-temporally appropriate for this developmental stage, were evident (Yagami *et al*, 1999). Engraftment of MM cells within the BM of injected embryos is demonstrated in Figure 1E and F. In the xenografted marrows, clones of myeloma cells surrounded by poor and aberrant haematopoiesis were observed. The sinus borders were not well defined, but chondrogenesis appeared to be normal in the affected bones (inset in Figure 1E). Reduced haematopoiesis in the BM of embryos injected with MM cells was confirmed by quantitative image analysis (Supplementary Figure 2S). The spleens of injected animals were not enlarged in contrast to the splenomegaly previously observed in embryos injected with leukaemia cells (Grinberg *et al*, 2009), although they were consistently redder (Figure 1G). Splenomegaly was not expected, as the spleen is not a major target organ for MM growth. Growths also formed on the CAM, which contained dividing CAG cells (Supplementary Figure 3S). At 1 week after injection, it continued to grow in size (Figure 1H).

### Myeloma cell engraftment in the BM

**Figure 2:**
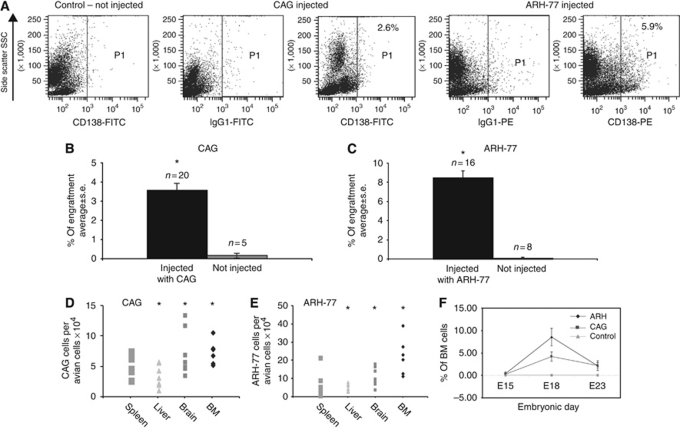
Engraftment of myeloma cells in embryonic turkey tissues. Cell suspensions from embryos injected with ARH-77 or CAG cells were incubated with fluorescein isothiocyanate (FITC) anti-human CD138 and allophycocyanin (APC) anti-human CD19. (**A**–**C**) Engraftment of human multiple myeloma (MM) cells in the bone marrow by flow cytometry. (**A**) A typical fluorescence activated cell sorter (FACS) analysis performed for the detection of human MM cells using anti-CD138 antibodies. (**B** and **C**) The average engraftment of cell lines. (**D** and **E**) Quantitative real-time polymerase chain reaction (Q-PCR) detection of human cells in embryonic organs presented as the amount of human alpha satellite DNA from embryos injected with CAG (**D**) and ARH-77 (**E**) compared with avian glyceraldehyde-3-phosphate dehydrogenase (*GAPDH*). Asterisks in (**A**)–(**E**) indicate *P*<0.005 compared with control embryos (Student's *t*-test). (**F**) Kinetics of engraftment from embryos killed on embryonic days (E)15, E18 and E23. Single-cell suspensions were prepared from the pooled bone marrow (BM) of two femurs from each embryo.

The engraftment was assessed 1 week after injection (on E11) of 5 × 10^6^ cells via the CAM veins as described previously (Grinberg *et al*, 2009). The kinetics of engraftment of MM cells were studied by screening for the presence of human MM cells on E15, E18, E21 and E23 in BM and other organs. The numbers of embryos tested and the different organs that were screened by FC analysis of BM, immunohistochemistry of CAM tissue and Q-PCR are detailed in the Supplementary Table 1S. Multiple myeloma cells in the developing embryonic BM were detected by FC. Figure 2A demonstrates the labelling of MM cells, CAG cells (middle panel) with FITC anti-CD138 and ARH-77 cells (right panel) with PE anti-CD138 in a typical BM sample. Flow cytometry analysis detected an average engraftment (±s.d.) of 3.5±0.38% CAG cells (*n*=20) and 8.5±0.17% ARH-77 (*n*=16) cells in the embryonic turkey BM (Figure 2B and C). Fewer than 0.5% of the myeloma cells were also detectable by FACS in the embryonic spleens (data not shown). Although FC was very useful for the analysis of BM, engraftment in other organs required molecular methods of detection. We therefore used Q-PCR real time to detect human myeloma cell engraftment in haematopoietic and non-haematopoietic tissue. The amount of genomic human alpha satellite DNA was compared with the amount of avian *GAPDH* in each of the organs tested. These included the haematopoietic organs BM, spleen and liver, as well as non-haematopoietic brain tissue. A significant amount of engraftment was also detected by Q-PCR in the BM as well as in the liver and brain of both cell lines compared with controls (*P*<0.005 by Student's *t*test) (Figure 2D and E). As expected, no MM cells were detected in the blood. The kinetics of BM engraftment of both cell lines determined by FC is depicted in Figure 2F. Few or no cells were detected at E15, which was 4 days after injection; however, the embryonic BM was very scanty on E15 and avian cells had not yet been well developed. The peak of human cell xenograft in the BM was observed on E18 and declined by E23 as illustrated in Figure 2F and detailed in Supplementary Table 1S.

### Detection of circulating human free immunoglobulin light chain in xenografted embryos

Multiple myeloma is characterised as a monoclonal plasma cell proliferative disorder. Testing the immunoglobulin FLC level (*λ* and *κ*) in the patients’ serum is of major clinical importance in diagnosis, prognosis and management of this disease (Dispenzieri *et al*, 2009). We adapted the FLC assay to detect human immunoglobulin in the serum of the E18 embryos after xenografting. A calibration curve was set up by mixing known levels of human *κ* FLC with E18 embryo serum and comparing it to human controls. The correlation was excellent (*R*=0.89), and this enabled systemic monitoring of engraftment by utilising FLC levels in embryo serum. Higher than normal ranges of *κ* light chain were identified clearly. All control embryos had values below the detection range of <5.4 mg l^−1^. The elevated FLC level in individual embryos was generally related to the engraftment level detected in the BM by FC (Table 2). The discrepancy observed in some embryos was expected since BM samples represent the femur content only while serum levels represent the amount of systemic circulating FLC.

### Xenografts of MM patient cells

The engraftment efficiency of patient samples was tested to develop the turkey embryo model for patient cell screening. Cells from MM patients having an 18–85% range of detectable disease were injected on day E11. Similar to our previous reports on leukaemia cells (Grinberg *et al*, 2009), 80% of the MM patient samples engrafted (Table 3), a value as good as the engraftment of patient MM samples reported in SCID mice (Tassone *et al*, 2005). Our ethics committee approved the use only of cells from routine BM aspirates of 1–2 ml, after all the necessary material was sent and not needed for clinical testing. Given that a xenograft requires 10^7^ MM cells per embryo, such numbers are not usually available from the remaining sample. In the samples that we could test, both fresh samples 2/2 engrafted, whereas the frozen or cultured cells did not consistently engraft. More experiments with fresh MM cells are needed to confirm this finding, and we are in the process of obtaining samples from other institutions.

### Drug treatment efficacy in xenografted turkey embryos

To investigate the utility of this model to screen drug activity *in vivo*, we tested three newly approved drugs known for their anti-myeloma activity, thalidomide, bortezomib and lenalinomide. The drugs were prescreened by XTT assays *in vitro* to confirm their toxicity on the cell lines used in this study. Following assessment of *in-vitro* activity, the drugs were injected *in vivo* on day E13, 2 days after injection of the MM cells. Before testing the activity of the drugs on MM engraftment, different concentrations of the drugs were injected to determine the lethal dose, 50% (LD 50) in the embryos and to examine the toxicity induced in the developing embryonic BM. Thalidomide was found to be too toxic to the developing embryos and could not be used to treat MM in this model. Bortezomib and lenalinomide at doses that were not BM toxic proved to be highly effective at eradicating the myeloma 1 week after treatment. Drug ranges were calculated from the therapeutic doses of the drugs for adult humans with an average blood volume of 5 l and for the turkey embryo with a volume of 3 ml. The equivalent therapeutic dose for thalidomide (100–200 mg dose per human adult) translates to 60–120 *μ*g per embryo. Concentrations as low as 0.12 *μ*g per embryo were lethal to all embryos injected (Table 4). We therefore abandoned the use of thalidomide in this system.

**Figure 3:**
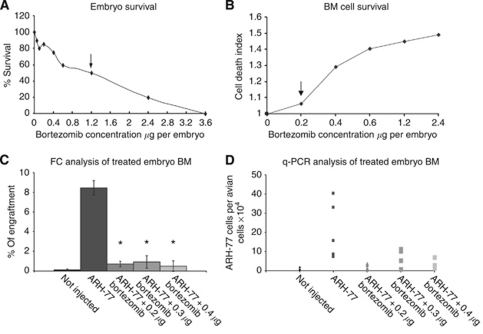
Bortezomib inhibits xenografted human myeloma cells in turkey embryos. Toxicity of different concentrations that were similar to therapeutic concentrations in patients was tested on embryonic day 13 (E13). (**A**) The lethal dose, 50% (LD 50) of bortezomib was 1.2 *μ*g per embryo as indicated by the arrow. (**B**) The highest non-toxic to bone marrow (BM) drug dose was 0.2 *μ*g per embryo as indicated by arrow. (**C**) Flow cytometry analysis of BM cells on day E13 of embryos injected with multiple marrow (MM) cells on E11 and different concentrations of bortezomib on E13 and cells harvested on day E18. (**D**) Quantitative real-time polymerase chain reaction (Q-PCR) quantification of the proportion of human cells in the BM of embryos treated with different concentrations of the drug. ^*^P⩽0.005 compared with untreated embryos (Student's *t*-test).

To test both LD 50 and toxicity to the developing BM of bortezomib, we injected increasing concentrations of 0.2–3.6 *μ*g per turkey embryo on E13. This range was calculated from the adult human therapeutic dose of 1.2–2 mg daily, which translates into a dose of 0.8–1.2 *μ*g per embryo. These concentrations correlated exactly to the LD 50 levels in the embryos (Table 5 and Figure 3A). We further reduced the doses of bortezomib to determine the viability of cells within the developing BM. Bone marrow cells from treated embryos were analyzed by FC using 7AAD staining of dead cells. Drug doses that did not increase the proportion of dead cells in the BM compared with untreated controls were chosen. Bortezomib at 0.2–0.3 *μ*g per embryo was found to be the highest dose that did not cause significant BM toxicity (Figure 3B). This concentration was used on day E13 to treat embryos that were previously injected with 5 × 10^6^ ARH-77 cells per embryo on E11. The engraftment was dramatically inhibited in the bortezomib-treated turkey embryos. This result was demonstrated by FC analysis (Figure 3C) and confirmed by Q-PCR of human alpha satellite DNA in BM tissue (Figure 3D). It was revealed that the engraftment was reduced from 8.5 to 0.72% in the ARH-77 cell line after treatment by FACS analysis. The results of Q-PCR analysis clearly showed a 16.5-fold reduction of human MM cells in the bortezomib-treated turkey embryos *vs* untreated embryos.

**Figure 4:**
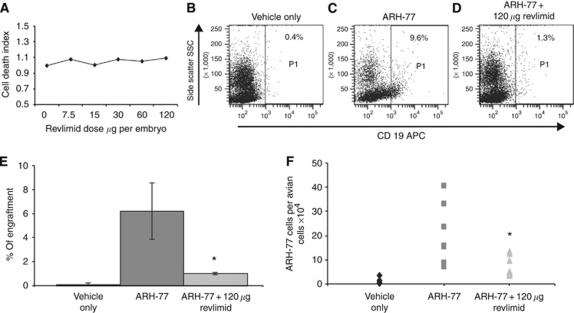
Efficacy of lenalinomide (Revlimid) treatment of multiple myeloma (MM)-injected turkey embryos. (**A**) Different concentrations of lenalinomide were injected into turkey embryos on embryonic day 13 (E13) and bone marrow (BM) cell toxicity was assessed. No toxicity was observed up to 120 *μ*g per embryo. ARH-77 cells were injected intravenously on E11, and embryos were treated with lenalinomide on E13. (**B**–**D)** Turkey embryos on E18. Bone marrow cells were stained with allophycocyanin (APC)-anti-human CD19. (**B**) A typical analysis of a vehicle-injected embryo. (**C**) An embryo xenografted with MM cells, and (**D**) a xenografted embryo treated with lenalinomide 120 *μ*g per embryo. (**E**) The average engraftment when analyzed by flow cytometry or by Q-PCR (**F**). ^*^P⩽0.005 compared with ARH-77-injected embryos (Student's *t*-test).

Experiments were also performed using lenalinomide, which is an immunomodulatory agent, similar to thalidomide, but less toxic and less teratogenic. The adult lenalinomide dose is around 25 mg per day, which translates into 15 *μ*g per embryo. Lenalinomide proved to be non-toxic to the embryos, and there was no evidence of LD 50. The survival rate of the embryos on E18 was 83% even at concentrations as high as 120 *μ*g per embryo (Table 6 and Figure 4A). The grafted MM cells were sensitive to a single dose of 120 *μ*g lenalinomide per embryo administered on E13 (2 days after the introduction of the cells). Flow cytometry analysis showed an 80% reduction in tumour load with no significant toxicity to the embryos (Figure 4A–E). Molecular detection of human alpha satellite DNA showed a 50% reduction in human cells (Figure 4F).

**Figure 5:**
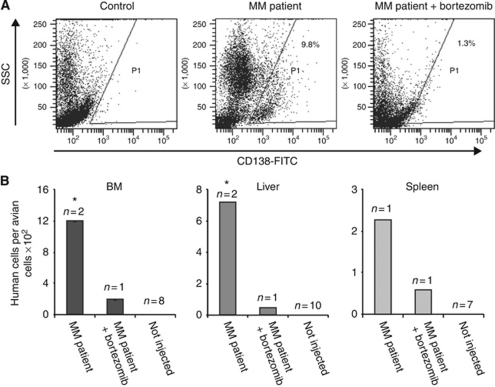
Bortezomib treatment of engrafted fresh human multiple myeloma (MM) in turkey embryos. (**A**) Flow cytometry detection of human MM cell engraftment into turkey embryo bone marrow (BM) and of bortezomib-treated embryo on embryonic day 13 (E13). Bone marrow from turkey embryos was collected on E18 and cells stained with fluorescein isothiocyanate (FITC) anti-human CD138 and APC anti-human CD19. Human cells were detected in the samples from injected embryos, but not in non-injected embryos. (**B**) Quantitative real-time polymerase chain reaction (Q-PCR) of human alpha satellite DNA also detected human cellular DNA in the turkey embryo haematopoietic organs. Human cells were detected in the samples from MM patient-injected embryos, but not in those treated by bortezomib. ^*^P⩽0.002 relative to MM patient-injected embryos.

Two samples of fresh MM cells (10^7^) from the BM of patients with >50% disease in their BM were injected on E11 and treated as described above with bortezomib 0.2 *μ*g per embryo on E13. Bone marrow from injected and non-injected turkey embryos was collected on E18, and MM cell engraftment was monitored by FC. The cells were stained with anti-human CD138 and CD38 as positive markers to determine engraftment. CD56 and CD45 were negative on the MM cells in all patient BMs tested and were used as negative control markers. An example of patient cells detected by FC with anti-CD138 in the BM of injected embryos is illustrated in Figure 5A. Multiple myeloma cells were also detected in the liver and spleen samples by Q-PCR. Administration of a single dose of bortezomib at a concentration that was demonstrated to be non-toxic to the BM eradicated MM cell growth in the embryonic BM (Figure 5B and C).

## Discussion

The development of therapeutic agents for MM could be facilitated by more rapid and less expensive *in-vivo* systems for evaluating drug activity drugs than the ones currently available. There are few *in-vivo* systems for the preclinical evaluation of new anti-myeloma therapy. The best current models are highly immune-deficient mice (Yaccoby *et al*, 1998; Tassone *et al*, 2005; Wunderlich *et al*, 2010). This study describes a novel xenograft model for studying MM that involves pre-immune turkey embryos and is rapid, low cost and easy to manipulate. Transplantation of cells and tissues to the embryonic chick has long been used to study the growth, spread and angiogenic potential of solid mammalian tumours, but it was never applied to the study of MM.

We had recently shown that the turkey embryo is a superior host than the chick embryo for xenografts of human blood malignancies, including two MM lines, CAG and U266 (Grinberg *et al*, 2009). In this study, we extended these observations by demonstrating the kinetics of MM engraftment, patient MM cell engraftment and the efficacy of modern chemotherapy drugs in killing MM in the turkey embryo. Preliminary experiments revealed that the optimal timing for intravenous injection of MM is on E11, when veins can easily be manipulated, and that the optimal time for engraftment is 7–9 days (data not shown). Engraftment of cell lines CAG and ARH-77 in grafted embryos is robust, as detected by both FC and RT–PCR. The embryonic BM cavities are just beginning to form during the first days after injection. Clearly delineated haematopoietic sinuses and robust haematopoiesis were observed within the BM of normally developing embryos at E18: all stages of erythropoiesis as well as prominent heterophils (avian granulocytes, lymphocytes and stromal cells could be seen). The xenografted embryos with growing myeloma, however, appeared to have fewer haematopoietic centres, with poorly defined sinus borders, implying that the MM cells were causing aberrant haematopoiesis. The human free *κ* light chain is easily detected in the blood of almost all the xenografted embryos, and its level is generally related to the engraftment level detected in the BM by FC. Free light chain levels, such as those measured in patients, provide an excellent readout of the tumour load and may be used in the future to determine the efficacy of drug treatment. The level of MM engraftment detected by FC and the FLC level were not correlated statistically, which is not surprising since the FC analysis was carried out on engraftment in the femur, while circulating FLC represents the systemic level of engraftment in all bones and in haematopoietic and non-haematopoietic compartments.

We also injected MM cells from patient BM samples. About 80% of the injected patient MM samples were engrafted, as determined by FC and Q-PCR, which is similar to the engraftment rates obtained with patient samples in NOD/SCID transplantation (Feo-Zuppardi *et al*, 1992) and similar to our previous reports on patient leukaemia cells injected in turkey embryos (Grinberg *et al*, 2009). Although the human-to-mouse xenograft is the most commonly used *in-vivo* system for studying leukaemia and myeloma, most patient samples do not adequately engraft in immune-deficient mice. The reasons for this remain unclear, but they could include homing and survival mechanisms in the foreign host, lack of appropriate human stromal cells and local growth factors in the murine marrow niche, and possibly innate immunity in the mice, as well as intrinsic diversity among leukaemia samples (Wunderlich *et al*, 2010). It is known that inherent fundamental variance exists between clinical samples of human leukaemias and their model cell lines. Gene ontology analyses demonstrated that genes related to DNA or RNA metabolism and genes related to macromolecule synthesis are highly active in leukaemia cell lines, and that genes related to immune or host response are overexpressed in clinical leukaemia samples (Leupin *et al*, 2006). This difficulty in obtaining better engraftment of patient samples was overcome in the SCID model by injecting patient MM cells directly into the bone (Yaccoby *et al*, 1998), a technique that is not possible in the turkey embryo model as the marrow cavity is not developed on E11, the optimal day of injection.

We have previously provided evidence that the avian embryo model can be used for testing the utility of chemotherapy against xenografted leukaemia by showing that doxorubicin dramatically reduced the engraftment of K562 and DAMI leukaemia lines (Grinberg *et al*, 2009). In this study, we demonstrate that the avian embryo model can be used to test the efficacy of anti-MM agents *in vivo*. Three newly FDA-approved drugs, thalidomide, bortezomib and lenalinomide, were tested for their anti-myeloma activity in this model. The drugs were injected intravenously 2 days after the grafting of MM cells, allowing the cells to home to and engraft in the haematopoietic organs. Different concentrations of the drugs were pretested to determine the LD 50 in the embryos and to examine their toxicity on the developing embryonic BM. Stringent testing of drug toxicity to the embryos and to their BM is essential since developing embryos are very sensitive owing to their highly proliferative growing tissues, including blood vessels in their BM. Indeed, thalidomide, a known teratogen (Ito *et al*, 2010), was found to be highly toxic to the embryos at very low levels and could not be used to treat MM in this model. The anti-myeloma activity of thalidomide is thought to be via its potent antiangiogenic properties, and it is possible that it killed the embryos by preventing sufficient blood vessel development.

We also tested bortezomib, a reversible, highly selective and potent inhibitor of the 26S proteasome, and lenalinomide, a thalidomide analogue with immunomodulatory and antiangiogenic properties. These newly FDA-approved first-line therapy drugs had lower toxicity and successfully treated MM in the turkey embryos. A single dose of these drugs administered at concentrations comparable to or lower than the human therapeutic dose were not toxic to the embryos or to their BM and dramatically reduced MM cell engraftment in the turkey embryos. Although few data were generated to demonstrate the feasibility of testing anti-myeloma drugs on fresh patient samples *in vivo*, these experiments nevertheless establish proof of principle that this is a feasible approach.

Both the SCID and avian embryo xenografts models can be used to further our understanding of the biology of MM and to test potential therapeutics. There are, however, several restrictions in using such *in-vivo* systems. The number of MM cells is limited in the BM samples harvested from an individual patient, thus limiting the number of embryos or mice that can be injected with cells from a single patient. Moreover, there is high variability in the time to engraftment in mice (i.e., 2–19 weeks) among patient samples. (Yaccoby *et al*, 1998; Tassone *et al*, 2005). With the turkey model, the engraftment window of 7–9 days, and some patient samples may require extended times, which are not possible in this rapid model. Additional limitations are that we do not know if the disease is recapitulated in the avian embryo model as it is in the mouse model, due to the short over all engraftment period. Tassone *et al* (2005) noted that only a fraction of engrafted specimens produce measurable or comparable paraprotein or osteolytic lesions in mice. At the present time, we do not know the exact nature of engrafting cells nor whether they can actually recapitulate disease. One direction for future experimental studies would be to compare the robustness of engraftment of patient samples in the new avian model to their engraftment in immune-deficient mice.

In conclusion, we have shown that a turkey embryo xenografted with CAG or ARH-77 cells provides a reproducible and predictable *in-vivo* model for human MM cell growth in the BM, thereby enabling the evaluation of new anti-MM drugs. This new MM model may facilitate faster *in-vivo* screening of anti-MM agents, thereby reducing drug development time and cost. With further development, the system may be made sufficiently consistent for testing therapeutics against patient samples and may provide a venue for personalised testing of drug sensitivity in relapsed refractory or in patients harbouring high-risk disease.

## Figures and Tables

**Table 1:** Primers for detecting and quantifying human and turkey cells using PCR

**Gene**	**Name**	**5′ → 3′ sequence**
*Human alpha satellite*	Cr17_1a^a^	GGGATAATTTCAGCTGACTAAACAG
	Cr17_1b^b^	TTCCGTTTAGTTAGGTGCAGTTATC
	Cr17_4b^c^	AAACGTCCACTTGCAGATTCTAG
*Avian GAPDH*	Forward	GAGGAAAGGTCGCCTGGTGGATCG
	Reverse	GGTGAGGACAAGCAGTGAGGAACG

Abbreviation: PCR=polymerase chain reaction.

a Forward primer.

b Reverse primer for conventional PCR.

c Reverse primer for quantitative PCR.

**Table 2:** Detection of malignant melanoma engraftment

**% CAG cells detected in BM by FC**	**FLC (mg l^−1^** **) detected in serum of CAG xenografted turkey embryos**	**% ARH-77 cells detected in BM by FC**	**FLC (mg l^−1^** **) detected in serum of ARH-77 xenografted turkey embryos**
1.4	6.77	8.5	7.15
4.7	5.85	7.2	5.56
10.9	6.06	6.3	9.58
5.6	9.14	12.3	29.56
1.5	<3	14.2	32.56
2.6	<3	2.6	17.35
9.6	47.2	5.6	12.85
5.3	18.5	13.1	7.05
5.9	29.5	8.1	24.2
9.3	5.39	9.1	<3
5	4.39	7.5	7.08
3.9	6.08	8.2	<3
1.3	7.09	4.3	3.09
5.15±3.18	13.27±13.56	8.23±3.37	14.18±10.27

Abbreviations: BM=bone marrow; FC=flow cytometry; FLC=free light chain; MM=multiple myeloma.

MM cells (5 × 10^6^) were injected intravenously on E11 and BM and serum collected 1 week later on E18. Engraftment was detected by FC and values presented are average %±s.d. FLC levels were detected by nephelometry and all control embryos had values below the detection range of <3 mg l^−1^.

**Table 3:** Engraftment of fresh MM cells from samples of patient BM

**Patient number**	**Cell source**	**% Plasma cells in the BM^a^**	**Disease status**	**Number of cells injected**	**% of MM BM engraftment^a^**	**FLC (mg l^−1^) detected in serum**	**Patient Rx^b^**
1	Fresh BM	21	Relapse	10^7^	1.2 3.0 1.1	NA	Dexamethasone
2	Fresh BM	85	Relapse	10^7^	20 9.8	NA	Dexamethasone Melphalan
3	Frozen BM	74	Relapse	10^7^	None	NA	Dexamethasone Melphalan
4	Frozen BM	18	Progressing disease	10^7^	7.9	NA	Dexamethasone Thalidomide
5	Fresh plasma cells cultured for one week	36	Relapse	6 × 10^6^	None	NA	Vorinostat Bortezomib
6	Frozen BM	NA	Progressing disease	0.4 × 10^6^	0.8	3.2	Vincristine, adriamycin, dexamethasone
7	Frozen BM	NA	Newly diagnosed	2 × 10^6^	1.6	5	Untreated
8	Frozen BM	NA	Newly diagnosed	0.5 × 10^6^	1	6.9	Untreated
9	Frozen BM	NA	Newly diagnosed	3 × 10^6^	0.6	6	Untreated
10	Frozen BM	NA	Progressing disease	0.5 × 10^6^	1.3	4.8	Vincristine, adriamycin, dexamethasone
11	Frozen BM	NA	Relapse	1.5 × 10^6^	1.3	<3	Dexamethasone Melphalan
12	Frozen BM	42	Newly diagnosed	10^7^	1	<3	Untreated
13	Frozen BM	13	Relapse	10^6^	2.6	4	Dexamethasone Melphalan
14	Frozen BM	70	Relapse	10^7^	1.6 2 1.6 1.4 1.4	4 6.9 5.75 4.9 5.44	Dexamethasone

Abbreviations: BM=bone marrow; FITC=fluorescein isothiocyanate; FLC=free light chain; MM=multiple myeloma; NA=not available; PBS=phosphate-buffered saline; PE=phycoerythrin.

Cells in PBS (100 *μ*l) or PBS alone (100 *μ*l) were injected intravenously on E11 and femur BM samples were collected and prepared as single-cell suspensions on day E18. These samples were analysed by flow cytometry using FITC anti-CD138 and PE anti-CD38 antibodies, and FLC levels were detected by nephelometry. Control embryos (*n*=27) had values below the sensitivity range of 3 mg l^−1^.

a Percentage of plasma cells as determined by CD38/CD138 and flow cytometry.

b Rx within 3 months before cell collection. Three cell samples were taken from newly diagnosed untreated patients, and all other samples were from treated patients with relapse or progressive disease.

**Table 4:** Survival of embryos after treatment with varying concentrations of thalidomide

**Thalidomide concentration**	**Number of turkey embryos injected on E13**	**Number of turkey embryos that survived by E18**
0 (vehicle only 50% PBS+50% ethanol)	6	5
30 *μ*g per embryo	6	0
60 *μ*g per embryo	6	0
120 *μ*g per embryo	6	0
0 (vehicle only 50% PBS+50% ethanol)	6	4
3 *μ*g per embryo	6	0
6 *μ*g per embryo	6	0
12 *μ*g per embryo	6	0
0 (vehicle only 50% PBS+50% ethanol)	6	5
0.3 *μ*g per embryo	6	0
0.6 *μ*g per embryo	6	0
0.12 *μ*g per embryo	6	0

Abbreviation: PBS=phosphate-buffered solution.

The human therapeutic dose is equivalent to 60–120 *μ*g per embryo.

**Table 5:** Survival of turkey embryos after treatment with increasing concentrations of bortezomib

**Bortezomib concentration (*μ*g per embryo)**	**Number of turkey embryos treated**	**Number of surviving turkey embryos**	**% Survival**
0	5	5	100
0.1	5	4	80
0.2	7	6	87
0.4	8	6	75
0.6	5	3	60
1.2	14	7	50
2.4	15	3	20
3.6	12	0	0

The human therapeutic dose is equivalent to 0.8–1.2 *μ*g per embryo.

**Table 6:** Survival of embryos after treatment with increasing concentrations of lenalinomide

**Lenalinomide dose**	**Survival (%)**	**Number of embryos treated**	**Number of embryos that survived**
0 (only 70% ethanol+30% PBS)	100	6	6
7.5 *μ*g per embryo	83	6	5
15 *μ*g per embryo	100	6	6
30 *μ*g per embryo	83	6	5
0 (only 70% ethanol+30% PBS)	83	6	5
30 *μ*g per embryo	100	6	6
60 *μ*g per embryo	66	6	4
120 *μ*g per embryo	83	6	5

Abbreviations: BM=bone marrow; PBS=phosphate-buffered solution.

The human therapeutic dose is equivalent to 15 *μ*g per embryo. Doses of up to 120 *μ*g per embryo were not lethal or toxic to the BM.

## References

[bib1] Borset M, Hjertner O, Yaccoby S, Epstein J, Sanderson RD (2000) Syndecan-1 is targeted to the uropods of polarized myeloma cells where it promotes adhesion and sequesters heparin-binding proteins. Blood 96: 2528–2536 11001907

[bib2] Boulland JL, Halasi G, Kasumacic N, Glover JC (2010) Xenotransplantation of human stem cells into the chicken embryo. J Vis Exp (41): pii 2071, doi:10.3791/2071 20644515 10.3791/2071 PMC3144657

[bib3] Chauhan D, Singh AV, Aujay M, Kirk CJ, Bandi M, Ciccarelli B, Raje N, Richardson P, Anderson KC (2010) A novel orally active proteasome inhibitor ONX 0912 triggers *in vitro* and *in vivo* cytotoxicity in multiple myeloma. Blood 116: 4906–4915 20805366 10.1182/blood-2010-04-276626 PMC3321748

[bib4] Dalloul RA, Long JA, Zimin AV, Aslam L, Beal K, Ann Blomberg L, Bouffard P, Burt DW, Crasta O, Crooijmans RP, Cooper K, Coulombe RA, De S, Delany ME, Dodgson JB, Dong JJ, Evans C, Frederickson KM, Flicek P, Florea L, Folkerts O, Groenen MA, Harkins TT, Herrero J, Hoffmann S, Megens HJ, Jiang A, de Jong P, Kaiser P, Kim H, Kim KW, Kim S, Langenberger D, Lee MK, Lee T, Mane S, Marcais G, Marz M, McElroy AP, Modise T, Nefedov M, Notredame C, Paton IR, Payne WS, Pertea G, Prickett D, Puiu D, Qioa D, Raineri E, Ruffier M, Salzberg SL, Schatz MC, Scheuring C, Schmidt CJ, Schroeder S, Searle SM, Smith EJ, Smith J, Sonstegard TS, Stadler PF, Tafer H, Tu ZJ, Van Tassell CP, Vilella AJ, Williams KP, Yorke JA, Zhang L, Zhang HB, Zhang X, Zhang Y, Reed KM (2011) Multi-platform next-generation sequencing of the domestic turkey (*Meleagris gallopavo*): genome assembly and analysis. PLoS Biol 8: pii: e1000475 10.1371/journal.pbio.1000475 PMC2935454 20838655

[bib5] Dazzi F, Hasserjian R, Gordon MY, Boecklin F, Cotter F, Corbo M, Capelli D, Goldman JM (2000) Normal and chronic phase CML hematopoietic cells repopulate NOD/SCID bone marrow with different kinetics and cell lineage representation. Hematol J 1: 307–315 11920208 10.1038/sj.thj.6200051

[bib6] Dispenzieri A, Kyle R, Merlini G, Miguel JS, Ludwig H, Hajek R, Palumbo A, Jagannath S, Blade J, Lonial S, Dimopoulos M, Comenzo R, Einsele H, Barlogie B, Anderson K, Gertz M, Harousseau JL, Attal M, Tosi P, Sonneveld P, Boccadoro M, Morgan G, Richardson P, Sezer O, Mateos MV, Cavo M, Joshua D, Turesson I, Chen W, Shimizu K, Powles R, Rajkumar SV, Durie BG (2009) International Myeloma Working Group guidelines for serum-free light chain analysis in multiple myeloma and related disorders. Leukemia 23: 215–224 19020545 10.1038/leu.2008.307

[bib7] Feo-Zuppardi FJ, Taylor CW, Iwato K, Lopez MH, Grogan TM, Odeleye A, Hersh EM, Salmon SE (1992) Long-term engraftment of fresh human myeloma cells in SCID mice. Blood 80: 2843–2850 1450409

[bib8] Feuring-Buske M, Gerhard B, Cashman J, Humphries RK, Eaves CJ, Hogge DE (2003) Improved engraftment of human acute myeloid leukemia progenitor cells in beta 2-microglobulin-deficient NOD/SCID mice and in NOD/SCID mice transgenic for human growth factors. Leukemia 17: 760–763 12682634 10.1038/sj.leu.2402882

[bib9] Ghosh N, Ye X, Ferguson A, Huff CA, Borrello I (2011) Bortezomib and thalidomide, a steroid free regimen in newly diagnosed patients with multiple myeloma. Br J Haematol 152: 593–599 21241279 10.1111/j.1365-2141.2010.08534.x PMC3412295

[bib10] Grinberg I, Reis A, Ohana A, Taizi M, Cipok M, Tavor S, Rund D, Deutsch VR, Goldstein RS (2009) Engraftment of human blood malignancies to the turkey embryo: a robust new *in vivo* model. Leuk Res 33: 1417–1426 19297019 10.1016/j.leukres.2009.02.009

[bib11] Ito T, Ando H, Suzuki T, Ogura T, Hotta K, Imamura Y, Yamaguchi Y, Handa H (2010) Identification of a primary target of thalidomide teratogenicity. Science 327: 1345–1350 20223979 10.1126/science.1177319

[bib12] Kobayashi T, Koshida K, Endo Y, Imao T, Uchibayashi T, Sasaki T, Namiki M (1998) A chick embryo model for metastatic human prostate cancer. Eur Urol 34: 154–160 9693252 10.1159/000019702

[bib13] Koop S, MacDonald IC, Luzzi K, Schmidt EE, Morris VL, Grattan M, Khokha R, Chambers AF, Groom AC (1995) Fate of melanoma cells entering the microcirculation: over 80% survive and extravasate. Cancer Res 55: 2520–2523 7780961

[bib14] Kumar A, Hozo I, Wheatley K, Djulbegovic B (2011) Thalidomide versus bortezomib based regimens as first-line therapy for patients with multiple myeloma: a systematic review. Am J Hematol 86: 18–24 21120867 10.1002/ajh.21904

[bib15] Leupin N, Kuhn A, Hugli B, Grob TJ, Jaggi R, Tobler A, Delorenzi M, Fey MF (2006) Gene expression profiling reveals consistent differences between clinical samples of human leukaemias and their model cell lines. Br J Haematol 135: 520–523 17061979 10.1111/j.1365-2141.2006.06342.x PMC1654200

[bib16] Li M, Chen F, Clifton N, Sullivan DM, Dalton WS, Gabrilovich DI, Nefedova Y (2011) Combined inhibition of Notch signaling and Bcl-2/Bcl-xL results in synergistic antimyeloma effect. Mol Cancer Ther 9: 3200–3209 10.1158/1535-7163.MCT-10-0372 PMC3058809 21159606

[bib17] Lock RB, Liem N, Farnsworth ML, Milross CG, Xue C, Tajbakhsh M, Haber M, Norris MD, Marshall GM, Rice AM (2002) The nonobese diabetic/severe combined immunodeficient (NOD/SCID) mouse model of childhood acute lymphoblastic leukemia reveals intrinsic differences in biologic characteristics at diagnosis and relapse. Blood 99: 4100–4108 12010813 10.1182/blood.v99.11.4100

[bib18] Melchert M, List A (2007) The thalidomide saga. Int J Biochem Cell Biol 39: 1489–1499 17369076 10.1016/j.biocel.2007.01.022

[bib19] Nefedova Y, Sullivan DM, Bolick SC, Dalton WS, Gabrilovich DI (2008) Inhibition of Notch signaling induces apoptosis of myeloma cells and enhances sensitivity to chemotherapy. Blood 111: 2220–2229 18039953 10.1182/blood-2007-07-102632

[bib20] Palumbo A (2010) First-line treatment of elderly multiple myeloma patients. Clin Adv Hematol Oncol 8: 529–530 20966887

[bib21] Pearce DJ, Taussig D, Zibara K, Smith LL, Ridler CM, Preudhomme C, Young BD, Rohatiner AZ, Lister TA, Bonnet D (2006) AML engraftment in the NOD/SCID assay reflects the outcome of AML: implications for our understanding of the heterogeneity of AML. Blood 107: 1166–1173 16234360 10.1182/blood-2005-06-2325 PMC1895911

[bib22] Peled A, Hardan I, Trakhtenbrot L, Gur E, Magid M, Darash-Yahana M, Cohen N, Grabovsky V, Franitza S, Kollet O, Lider O, Alon R, Rechavi G, Lapidot T (2002) Immature leukemic CD34+CXCR4+ cells from CML patients have lower integrin-dependent migration and adhesion in response to the chemokine SDF-1. Stem Cells 20: 259–266 12004084 10.1634/stemcells.20-3-259

[bib23] Rebbaa A, Chu F, Sudha T, Gallati C, Dier U, Dyskin E, Yalcin M, Bianchini C, Shaker O, Mousa SA (2009) The anti-angiogenic activity of NSITC, a specific cathepsin L inhibitor. Anticancer Res 29: 4473–4481 20032394

[bib24] Ribatti D, Nico B, Pezzolo A, Vacca A, Meazza R, Cinti R, Carlini B, Parodi F, Pistoia V, Corrias MV (2006) Angiogenesis in a human neuroblastoma xenograft model: mechanisms and inhibition by tumour-derived interferon-gamma. Br J Cancer 94: 1845–1852 16721359 10.1038/sj.bjc.6603186 PMC2361332

[bib25] Richardson P, Mitsiades C, Laubach J, Schlossman R, Ghobrial I, Hideshima T, Munshi N, Anderson K (2010) Lenalidomide in multiple myeloma: an evidence-based review of its role in therapy. Core Evid 4: 215–245 20694078 10.2147/ce.s6002 PMC2899783

[bib26] Taizi M, Deutsch VR, Leitner A, Ohana A, Goldstein RS (2006) A novel and rapid *in vivo* system for testing therapeutics on human leukemias. Exp Hematol 34: 1698–1708 17157167 10.1016/j.exphem.2006.07.005

[bib27] Tassone P, Neri P, Carrasco DR, Burger R, Goldmacher VS, Fram R, Munshi V, Shammas MA, Catley L, Jacob GS, Venuta S, Anderson KC, Munshi NC (2005) A clinically relevant SCID-hu *in vivo* model of human multiple myeloma. Blood 106: 713–716 15817674 10.1182/blood-2005-01-0373 PMC1895174

[bib28] Wunderlich M, Chou FS, Link KA, Mizukawa B, Perry RL, Carroll M, Mulloy JC (2010) AML xenograft efficiency is significantly improved in NOD/SCID-IL2RG mice constitutively expressing human SCF, GM-CSF and IL-3. Leukemia 24: 1785–1788 20686503 10.1038/leu.2010.158 PMC5439963

[bib29] Yaccoby S, Barlogie B, Epstein J (1998) Primary myeloma cells growing in SCID-hu mice: a model for studying the biology and treatment of myeloma and its manifestations. Blood 92: 2908–2913 9763577

[bib30] Yagami K, Suh JY, Enomoto-Iwamoto M, Koyama E, Abrams WR, Shapiro IM, Pacifici M, Iwamoto M (1999) Matrix GLA protein is a developmental regulator of chondrocyte mineralization and, when constitutively expressed, blocks endochondral and intramembranous ossification in the limb. J Cell Biol 147: 1097–1108 10579728 10.1083/jcb.147.5.1097 PMC2169349

[bib31] Zhang QL, Wang L, Zhang YW, Jiang XX, Yang F, Wu WL, Janin A, Chen Z, Shen ZX, Chen SJ, Zhao WL (2009) The proteasome inhibitor bortezomib interacts synergistically with the histone deacetylase inhibitor suberoylanilide hydroxamic acid to induce T-leukemia/lymphoma cells apoptosis. Leukemia 23: 1507–1514 19282831 10.1038/leu.2009.41

