# Supplementary figures and images for: Rapid in vivo testing of drug response in multiple myeloma made possible by xenograft to turkey embryos
Source: Br J Cancer. 2011 Nov 1;105(11):1708–18. doi: 10.1038/bjc.2011.445 (PMC3242603; doi:10.1038/bjc.2011.445)

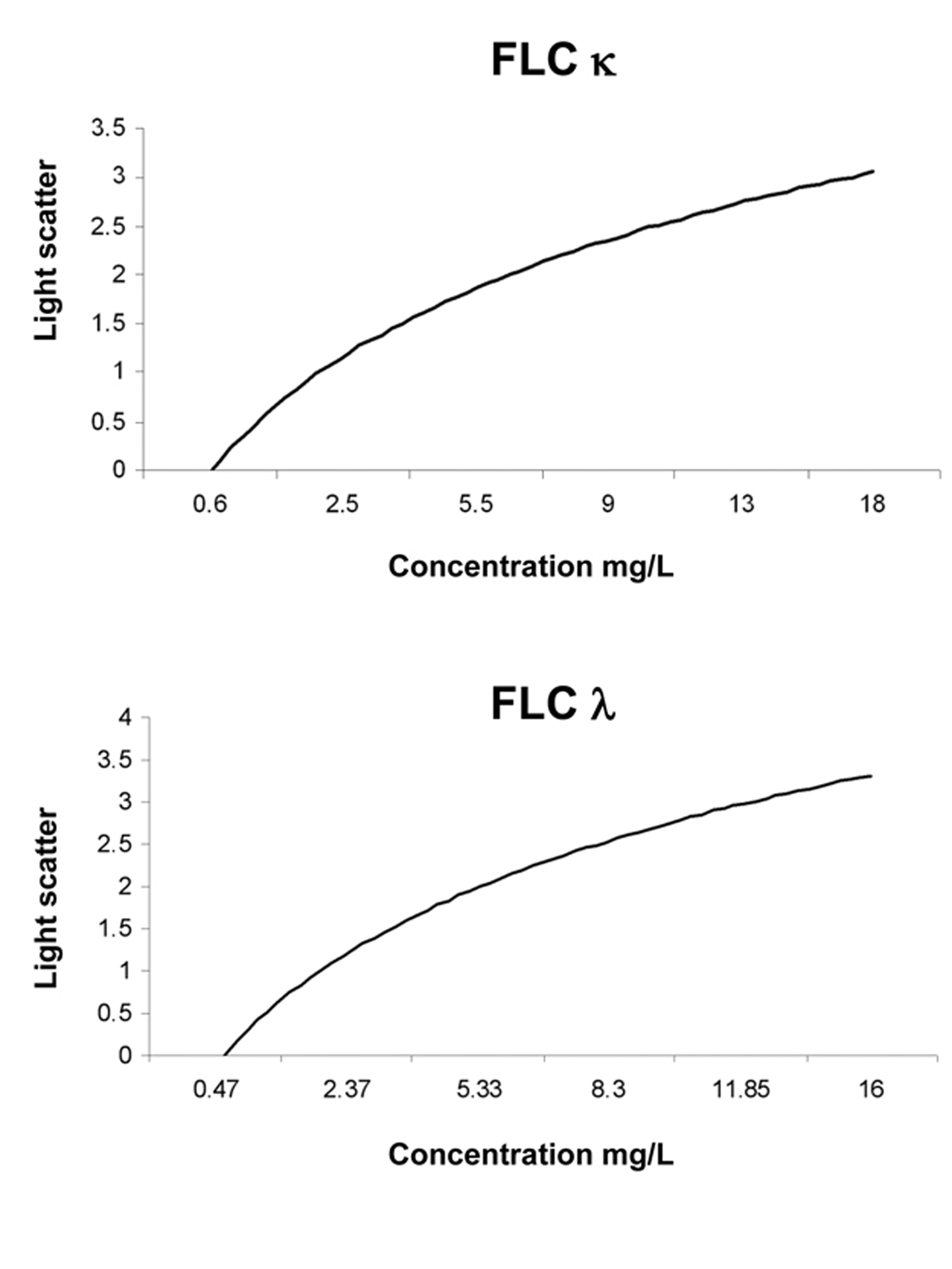

Supplement: Supplementary Figure S1 [file bjc2011445x1.tif]

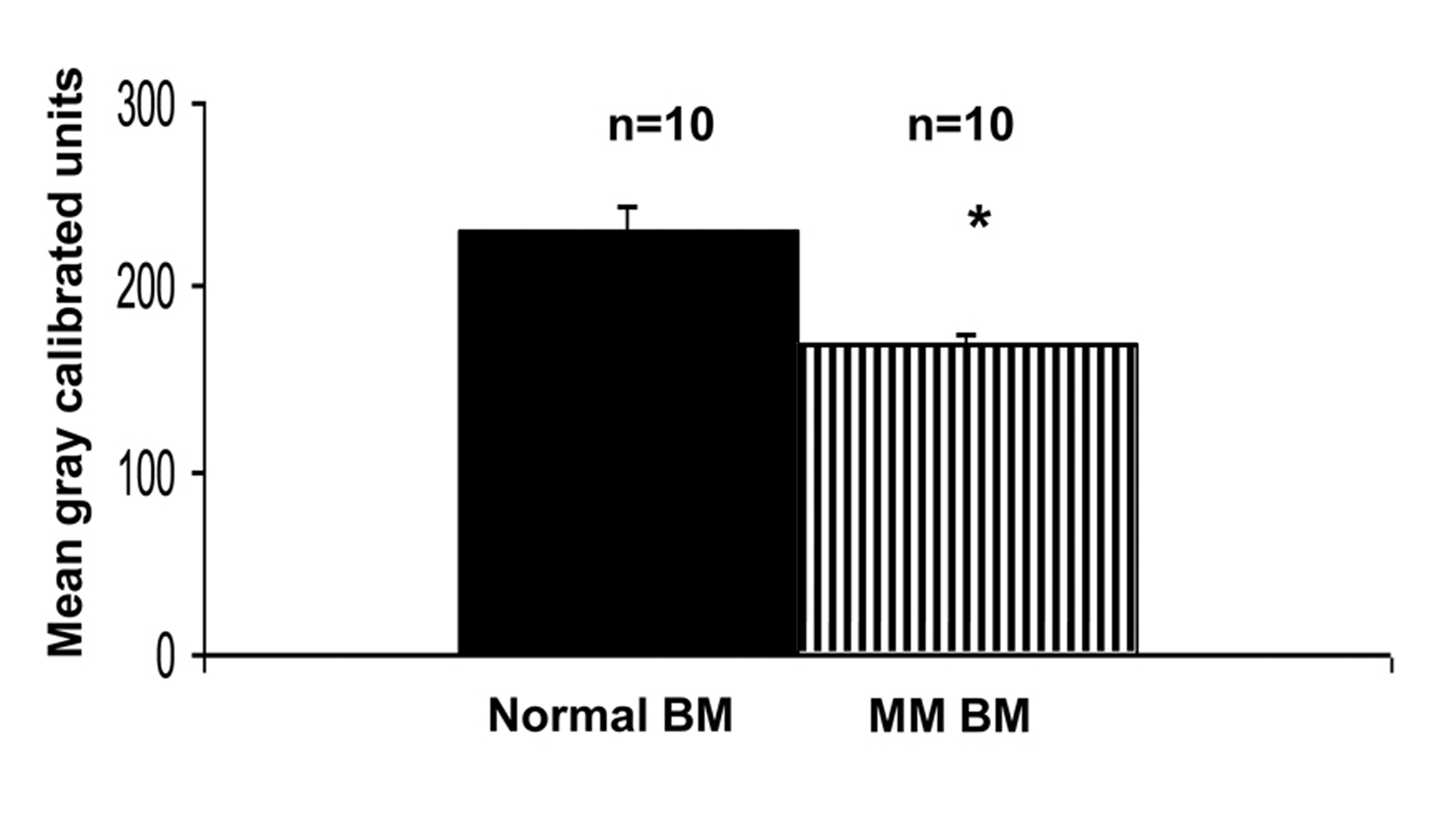

Supplement: Supplementary Figure S2 [file bjc2011445x2.tif]

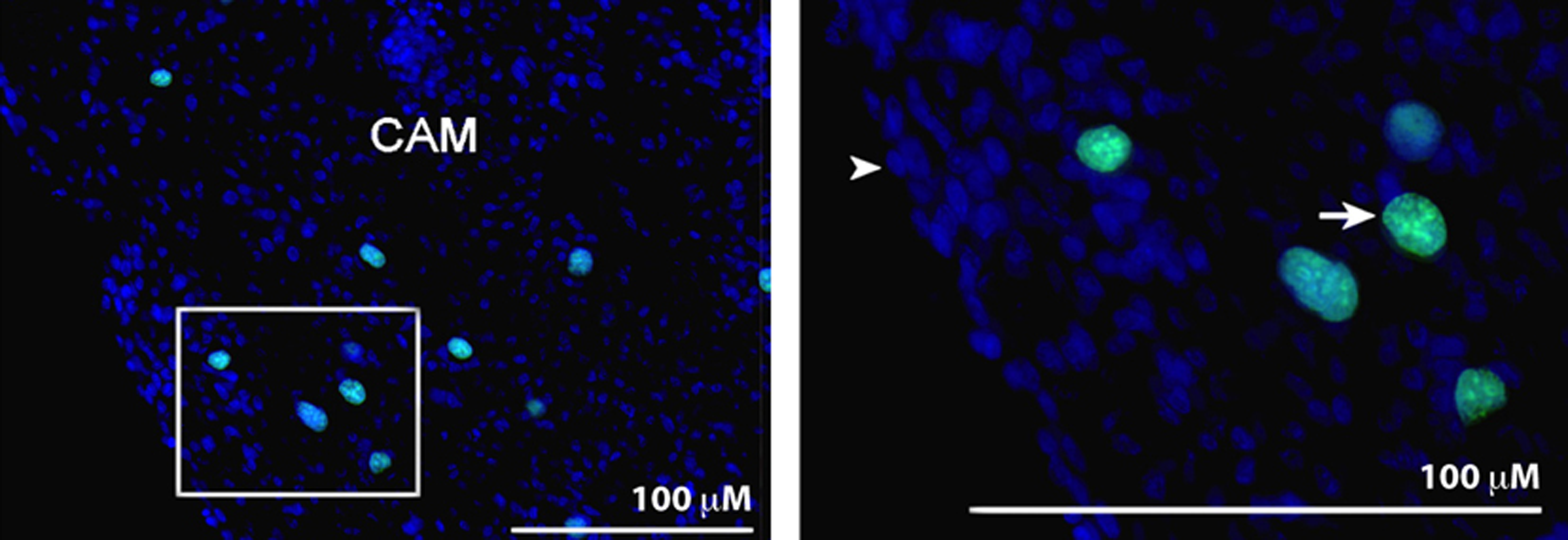

Supplement: Supplementary Figure S3 [file bjc2011445x3.tif]
