# Supplementary material for: Rapid in vivo testing of drug response in multiple myeloma made possible by xenograft to turkey embryos
Source: Br J Cancer. 2011 Nov 1;105(11):1708–18. doi: 10.1038/bjc.2011.445 (PMC3242603; doi:10.1038/bjc.2011.445)
Supplement: Supplementary Figure Legends [file bjc2011445x4.doc]

**Figure 1 S. Free Light Chain FLC calibration**

Circulating FLC κ or λ was performed by nephelometry using a turbidimetry assay. The reaction involves the formation of antigen antibody complexes, which increasingly scatter light causing a measurable decrease in intensity of the incident beam of light. FLC κ or λ concentration is proportional to the amount of immune complex formed. The graph represents the calibration curve generated from known concentrations of FLC (www.bindingsite.co.uk). The concentration of FLC in the test serum was calculated from a calibration curve. Commercially available controls were assayed as well as negative control sera from uninjected turkey embryos. The normal level in uninjected embryonic turkey serum was below the detection range of the instrument and determined to be < 3mg/L

(n= 27 ).

**Figure 2 S .** **Engraftment of MM cells in embryos reduces hematopoiesis in the developing bone marrow**

Paraffin sections of femur bones from embryos injected or not injected with human malignant myeloma cells were stained with hematoxylin and eosin (H&E).. Photomicrographs were taken using an Olympus BX60 microscope and a Scion digital camera (www.scioncorp.com) .Quantification of cells in hematopoietic niches in femur bones from turkey embryos performed by image analysis using Image J program (http://rsb.info.nih.gov/ij) Hematopoietic areas of similar sizes were delineated with Image-J, and scanned. The amount of cells within these areas was quantified using the mean gray scale value within these areas. As the images were colored the mean was calculated by converting each colored pixels (RGB) to grayscale using Image J formula. The mean gray value is reported in calibrated units.

**Figure 3 S . Injected human malignant myeloma (MM) cells engraft in the chorioallantoic membrane (CAM).**

Immunohistochemical staining of growth on CAM with Hoechst stain which labels all nuclei (small arrow) and FITC anti-human KI-67 (larger arrow) that labels only dividing human cells, at X10 original magnification (A) and at X40 original magnification of the area in the box (B). Most of the growth contained avian tissue (blue nuclei), yet the prominent presence of dividing human MM cells was easily detected (large green nuclei). Photomicrographs were taken on fluorescence-equipped Olympus BX60 microscope using Scion color digital cameras (www.scioncorp.com) and ImageJ (<http://rsb.info.nih.gov/ij/>) software. The scale bars represent 100 microns.
