# Supplementary material for: Rapid in vivo testing of drug response in multiple myeloma made possible by xenograft to turkey embryos
Source: Br J Cancer. 2011 Nov 1;105(11):1708–18. doi: 10.1038/bjc.2011.445 (PMC3242603; doi:10.1038/bjc.2011.445)
Supplement: Supplementary Table S1 [file bjc2011445x5.doc]

**Table 1 S** Samples tested to determine kinetics of myeloma cell engraftment

| **Day of detection** | **Organs harvested** | **Method of xenograft detection** | **Number of surviving control turkey embryos** | **Number of control turkey**  **embryos** | **Number of surviving injected turkey embryos** | **Number of injected turkey embryos** | **Cells injected** |
| --- | --- | --- | --- | --- | --- | --- | --- |
| E15 | BM, spleen, liver | FACS, real-time PCR | 6 | 6 | 10 | 10 | ARH-77 |
| E15 | BM, spleen liver | FACS, real-time PCR | 6 | 6 | 6 | 7 | CAG |
| E18 | BM, spleen,, liver, blood | FACS, real-time PCR | 8 | 8 | 25 | 26 | ARH-77 |
| E18 | BM, spleen, liver, blood | FACS, real-time PCR | 6 | 7 | 20 | 24 | CAG |
| E23 | BM, spleen, liver, blood | FACS, real-time PCR | 6 | 6 | 7 | 8 | ARH-77 |
| E21 | CAM | Immunohistochemistry real-time PCR | 3 | 3 | 3 | 3 | CAG |
| E21 | CAM | Immunohistochemistry real-time PCR | 4 | 4 | 4 | 4 | ARH-77 |
| E23 | BM, spleen liver, blood | FACS, real-time PCR | 6 | 6 | 7 | 8 | CAG |

Abbreviations: BM = bone marrow; PCR = polymerase chain reaction; CAM = chorioallantoic membrane.

MM cells (5X10^6^) of were injected IV via the CAM veins. Xenografts were detected at different time points in the indicated organs by FACS analysis of BM tissue, using CD19 or CD138 antibodies to detect MM cells.
